# Essential role of autoactivation circuitry on Aurora B-mediated H2AX-pS121 in mitosis

**DOI:** 10.1038/ncomms12059

**Published:** 2016-07-08

**Authors:** Midori Shimada, Takahiro Goshima, Hiromi Matsuo, Yoshikazu Johmura, Mayumi Haruta, Kazuhiro Murata, Hiromitsu Tanaka, Masahito Ikawa, Keiko Nakanishi, Makoto Nakanishi

**Affiliations:** 1 Department of Cell Biology, Graduate School of Medical Sciences, Nagoya City University, 1 Kawasumi, Mizuho-cho, Mizuho-ku, Nagoya 467-8601, Japan; 2 Faculty of Pharmaceutical Sciences, Nagasaki International University, Sasebo, Nagasaki 859-3298, Japan; 3 Research Institute for Microbial Diseases, Osaka University, Suita, Osaka 565-0871, Japan; 4 Department of Perinatology, Institute for Developmental Research, Aichi Human Service Center, 713-8 Kamiya-cho, Kasugai, Aichi 489-0392, Japan; 5 Division of Cancer Cell Biology, Department of Cancer Biology, Institute of Medical Science, The University of Tokyo, 4-6-1 Shirokanedai, Minato-ku, Tokyo 108-8639, Japan

## Abstract

Proper deposition and activation of Aurora B at the centromere is critical for faithful chromosome segregation in mammals. However, the mechanistic basis for abrupt Aurora B kinase activation at the centromere has not yet been fully understood. We demonstrate here that Aurora B-mediated phosphorylation of histone H2AX at serine 121 (H2AX-pS121) promotes Aurora B autophosphorylation and is essential for proper chromosome segregation. Aurora B-mediated H2AX-pS121 is specifically detected at the centromere during mitosis. H2AX depletion results in a severe defect in activation and deposition of Aurora B at this locus. A phosphomimic mutant of H2AX at S121 interacts with activated Aurora B more efficiently than wild-type *in vitro*. Taken together, these results propose a model in which Aurora B-mediated H2AX-pS121 probably provide a platform for Aurora B autoactivation circuitry at centromeres and thus play a pivotal role in proper chromosome segregation.

## Abstract (web summary)

Aurora B activation at the centromere is critical for faithful chromosome segregation in mammals. Here the authors show that Aurora B-mediated phosphorylation of histone H2AX at serine 121 is essential for Aurora B auto-activation circuitry at centromeres, ensuring proper chromosome segregation.

## Graphical abstract

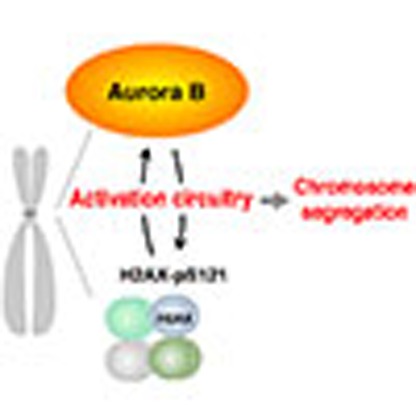

Successful chromosome segregation is coordinated by phosphorylation and dephosphorylation of a set of proteins that localize to chromosomes during mitosis^1^,^2^. The chromosomal passenger complex (CPC) is a highly conserved complex that orchestrates various mitotic events^3^,^4^,^5^. The CPC comprises Aurora B protein kinase and its non-enzymatic subunits, the inner centromere protein (INCENP), Survivin and Borealin. Aurora B kinase within the CPC plays a central role in triggering mitotic processes through phosphorylation of substantive substrates^6^,^7^,^8^,^9^. Therefore, the dynamic localization and activation of CPC should be rigorously regulated with respect to time and space, to ensure accurate chromosome segregation. In mammalian cells in late S phase, the CPC is first detected on pericentromeric heterochromatin through HP1 binding to INCENP^10^. Aurora B-mediated histone H3 phosphorylation at Ser10 (H3-pS10) then dissociates HP1 from histone H3 trimethylated lys9, leading to dissociation of the CPC from the chromosome arm^11^,^12^. Subsequently, the CPC accumulates at the inner centromere in early mitosis. This CPC accumulation at the centromere is partly achieved via the Haspin-H3-pT3 pathway^13^,^14^, which was shown to be boosted via the Bub1-H2A-Shugosin pathway^15^. Aurora B kinase activity itself is also essential for forcing CPC to localize to the inner centromere^16^,^17^, at least in part through a positive feedback loop between Haspin and Aurora B^18^. Aurora B kinase activity is rigorously regulated at multiple levels during cell cycle progression. Aurora B initially binds to the IN box of INCENP^19^, leading to low levels of Aurora B activation. This binding enables Aurora B to phosphorylate a carboxy-terminal Thr-Ser-Ser (TSS) motif in INCENP, further enhancing Aurora B kinase activity^20^,^21^. For full activation of Aurora B, phosphorylation of T232 in the T-loop of the Aurora B kinase domain is critically required^22^. This phosphorylation event is likely to occur in *trans*^23^, because increasing the local density of the CPC stimulates Aurora B activity^24^. However, the mechanism leading to the culminated and circumscribed activation of Aurora B at centromeres before activation of the Haspin-pT3 pathway has not been fully understood.

Histone H2AX is a variant of the H2A protein family and is a component of the histone octomer in nucleosomes^25^. Phosphorylation of H2AX by ataxia telangiectasia mutated (ATM) plays an important role in the DNA damage response by recruiting the various proteins involved in DNA repair and cell cycle checkpoints^26^,^27^, but little is known about other functions of this histone variant in normal cell cycle progression^28^. Here we report that Aurora B phosphorylates H2AX on S121 specifically on centromeres during mitosis, providing a platform for Aurora B autoactivation circuitry.

## Results

### H2AX is required for proper chromosome segregation

**Figure 1:**
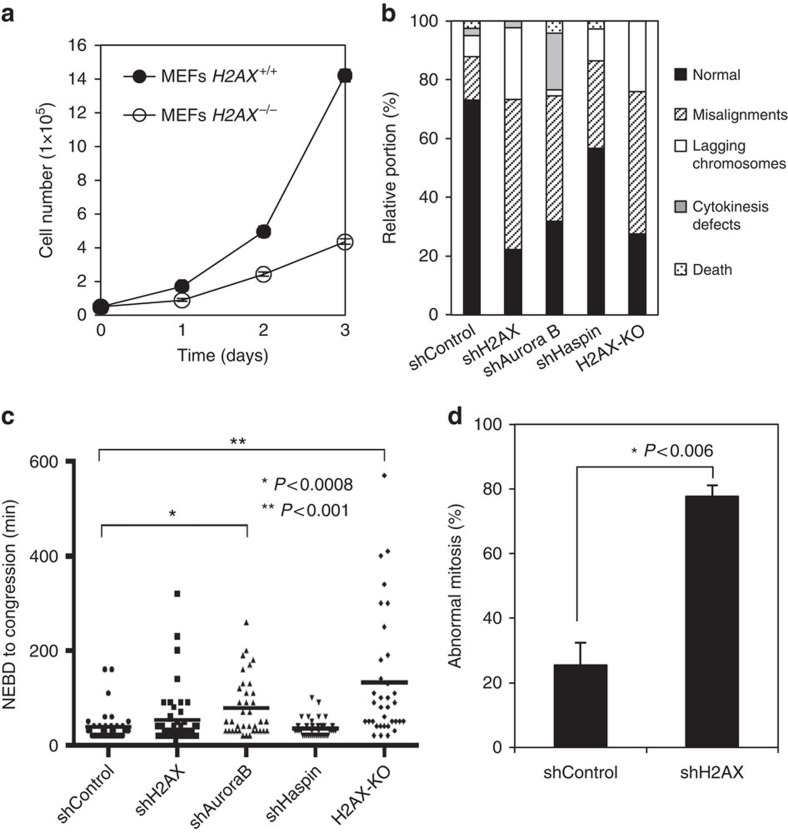
H2AX is required for proper chromosome segregation. (**a**) H2AX^+/+^ and H2AX^−/−^ MEFs were cultured and their cell numbers were counted at the indicated time points. Data are presented as means ±s.d. of at least three independent experiments. (**b**,**c**) HeLa cells with H2B-EGFP expressing the Dox-inducible shControl, shH2AX, shAurora B or shHaspin at 24 h after the addition of Dox or H2AX-KO HeLa cells with H2B-EGFP were subjected to time-lapse imaging for 24 h during cell division to monitor chromosome dynamics. Quantification of cells with the indicated phenotypes is shown (**b**) (*n*⩾35). Time interval between nuclear envelope breakdown (NEBD) and congression were measured (**c**) (*n*⩾35, Student's *t*-test). (**d**) HeLa cells with H2B-EGFP expressing the Dox-inducible shControl or shH2AX were incubated with doxycycline (1 μg ml^−1^) for 16 h and then synchronized at G2 phase by RO-3306 for 28 h. After release from RO, time-lapse imaging was performed for 4 h to detect mitotic defects. Percentages of aberrant mitoses are shown as means±s.d. of at least three independent experiments (*n*⩾20, Student's *t*-test).

Given that H2AX knockout (KO) mice are growth retarded^29^, we speculated that this H2A variant could function in normal cell cycle progression. H2AX^−/−^ mouse embryonic fibroblasts (MEFs) showed a severe defect in cell proliferation (Fig. 1a), as reported previously^29^. A sudden loss of H2AX through its short hairpin RNA (shRNA) also resulted in a defect in the proliferation of RPE-1 and HeLa cells (Supplementary Fig. 1a,b). As H2AX is involved in the maintenance of genome integrity and H2AX^−/−^ MEFs can be cultured for long periods, a defect in cell proliferation in H2AX^−/−^ MEFs might result from the accumulation of gene abnormalities that might affect cell cycle progression. We therefore generated H2AX KO HeLa cells using the CRISPR/Cas9 system (Supplementary Fig. 2a) and used them in further analyses. The complete loss of H2AX protein was confirmed by immunoblotting and preparing mitotic chromosomal spreads (Supplementary Fig. 2b,c). H2AX-KO HeLa cells also showed a defect in cell proliferation (see Supplementary Fig. 10b). To determine the reason for this defect, we performed time-lapse imaging analysis using both H2AX KO and H2AX knockdown (KD) HeLa cells harbouring H2B-EGFP, to visualize the chromatin structure. Surprisingly, we found that the majority of H2AX-KO and −KD HeLa cells showed gross abnormalities in mitosis, such as chromosome misalignment and lagging chromosomes (Fig. 1b). Similar types of mitotic abnormalities were also observed in Aurora B-depleted (shAurora B) and Haspin-depleted (shHaspin) HeLa cells, both kinases which are known to be essential for proper chromosome segregation (Fig. 1b), suggesting that the defects in the cell proliferation of H2AX-KD cells appeared to be due to mitotic abnormalities. Such abnormalities were not detected or only barely detected in control HeLa cells. Representative images of normal and abnormal mitoses were shown (Supplementary Movies 1–5). We detected a slight extension of the time interval between nuclear envelope breakdown and chromosome congression in H2AX-KO cells, although this extension was not detected in H2AX-KD cells (Fig. 1c). To examine whether mitotic abnormalities in H2AX-KD HeLa cells resulted from a DNA replication error due to an impaired DNA damage response, cells were synchronized at G2 phase by treatment with RO-3306 and H2AX was then depleted. H2AX-KD cells were released into mitosis by the removal of RO-3306. Time-lapse imaging revealed that H2AX depletion in HeLa cells markedly increased mitotic abnormalities when compared with control HeLa cells (Fig. 1d). Taken together, these results suggest the prerequisite role of H2AX in mitotic progression.

### Phosphorylation of H2AX at S121 in the centromeres

**Figure 2:**
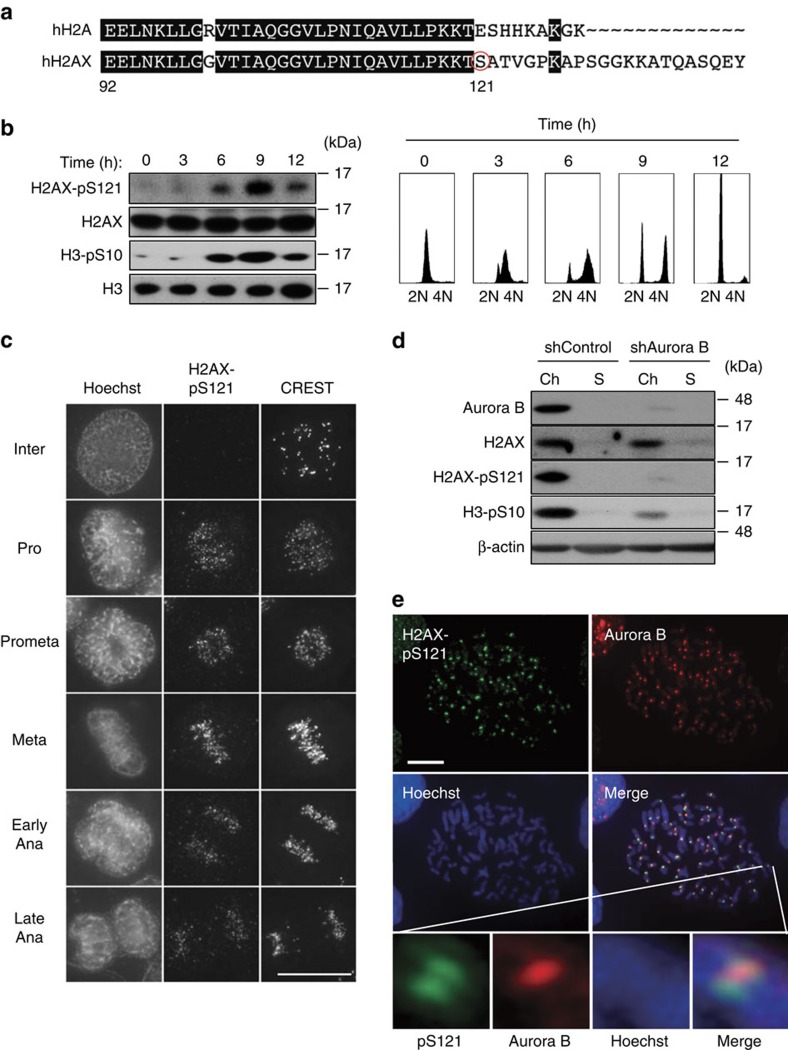
Mitotic phosphorylation of H2AX at S121 predominantly localizes at centromeres and is mediated by Aurora B. (**a**) Sequence alignment of C-terminal regions of *Homo sapiens* H2A and H2AX. Amino acids conserved in both histones are highlighted in black. S121 of H2AX is circled in red. (**b**) HeLa cells were synchronized by a double thymidine block and released into S phase. Cells were harvested at the indicated times and the lysates were subjected to immunoblotting with the indicated antibodies (left). Cell cycle profiles at the indicated times were monitored by FACS analysis (right). (**c**) Asynchronously growing HeLa cells were fixed and stained with anti-H2AX-pS121 and CREST antibodies. DNA was counterstained with Hoechst. Representative images captured during the cell cycle are shown. Scale bar, 10 μm. (**d**) HeLa cells expressing the Dox-inducible shControl or shAurora B were cultured in the presence of doxycycline (1 μg ml^−1^) for 3 days and treated with nocodazole (0.1 μg ml^−1^) for 12 h. After mitotic shake-off, chromatin (Ch) and soluble (S) fractions from mitotic cells were subjected to immunoblotting using the indicated antibodies. (**e**) Chromosome spreads were prepared from HeLa cells and stained with antibodies against H2AX-pS121 (green) and Aurora B (red). DNA was counterstained with Hoechst (blue). Prometaphase chromosomes and magnified images of paired sister chromatids are shown. Scale bar, 10 μm.

H2AX has a unique C-terminal domain and covalent modifications occur at this region (Fig. 2a [Fig f3]), such as phosphorylation at S139 (γH2AX) by ATM^30^,^31^, which is known to function as a platform for recruiting the various proteins involved in the DNA damage response to DNA damage sites^32^. Therefore, we speculated that this is also the case with the mitotic functioning of H2AX. An examination of a mitotic phosphorylation database revealed that H2AX-pS121 is markedly induced in mitosis (8.5-fold)^33^ and the residue around S121 was well matched with the Aurora B consensus phosphorylation site (R/K-X-S/T) (Fig. 2a). Thus, to determine the physiological relevance of this phosphorylation in mitosis, we generated phospho-specific polyclonal antibodies to this site. When HeLa cells were synchronized at G1/S phase by double thymidine treatment and then released into S phase, immunoblotting revealed that H2AX-pS121 was predominant during mitosis (Fig. 2b) and had similar kinetics to that of H3-pS10. We then determined the subcellular localization of H2AX-pS121 during mitosis. H2AX-pS121 was hardly detectable in interphase cells, but was detected on chromatin in mitotic cells (Supplementary Fig. 3a). Importantly, H2AX-pS121 predominantly co-localized with a centromere marker, CREST, on immunohistochemical stains and chromosomal spreads, indicating that H2AX at S121 was specifically phosphorylated at the centromere (Fig. 2c and Supplementary Fig. 3b). The specificity of this phospho-specific antibody was confirmed in several ways, showing that this antibody recognized neither the ectopically expressed S121A mutant nor endogenous mitotic H2AX treated with calf intestinal phosphatase (Supplementary Fig. 4a,b). Centromeric signals of H2AX-pS121 were not detected on either immunohistochemical staining or on chromosomal spreads of H2AX-KD or -KO HeLa cells (Supplementary Fig. 4c,d). Immunohistochemial analyses also revealed that this antibody failed to recognize mitotically condensed chromatins and those expressing S121A in H2AX^−/−^ MEFs (Supplementary Fig. 4e). We then tried to identify the kinase responsible for this phosphorylation using mitotic kinase inhibitors. An Aurora B inhibitor, ZM-447439, strongly suppressed H2AX-pS121 in nocodazole-treated cells, whereas a Plk1 inhibitor, BI2546, failed to do so (Supplementary Fig. 5a). Consistent with this, Aurora B depletion resulted in an almost complete loss of H2AX-pS121 in the chromatin fraction and centromere signals in the chromosome spreads (Figs 2d and 4e). Although H2AX-pS121 was detectable in condensed chromosomes during anaphase, these signals as well as H3-pT3 were lost in Aurora B-depleted cells, suggesting that Aurora B-mediated H2AX-pS121 is still present during anaphase (Supplementary Fig. 5b). An *in vitro* kinase assay using [^32^P] ATP revealed at least one additional site in H2AX phosphorylated by Aurora B (Supplementary Fig. 5c). Immunofluorescence analysis of nocodazole-arrested HeLa chromosome spreads revealed that Aurora B appeared not to completely co-localize but to partly overlapsed with H2AX-pS121 (Fig. 2e). A zoomed-in view of prometaphase chromosomes revealed that H2AX-pS121 signals localized along the two chromatid axes, and that these signals culminated near the kinetochores. In contrast, Aurora B predominantly localized to the intersister region as reported previously^15^.

### H2AX-pS121 plays a critical role in Aurora B autoactivation

**Figure 3 f3:**
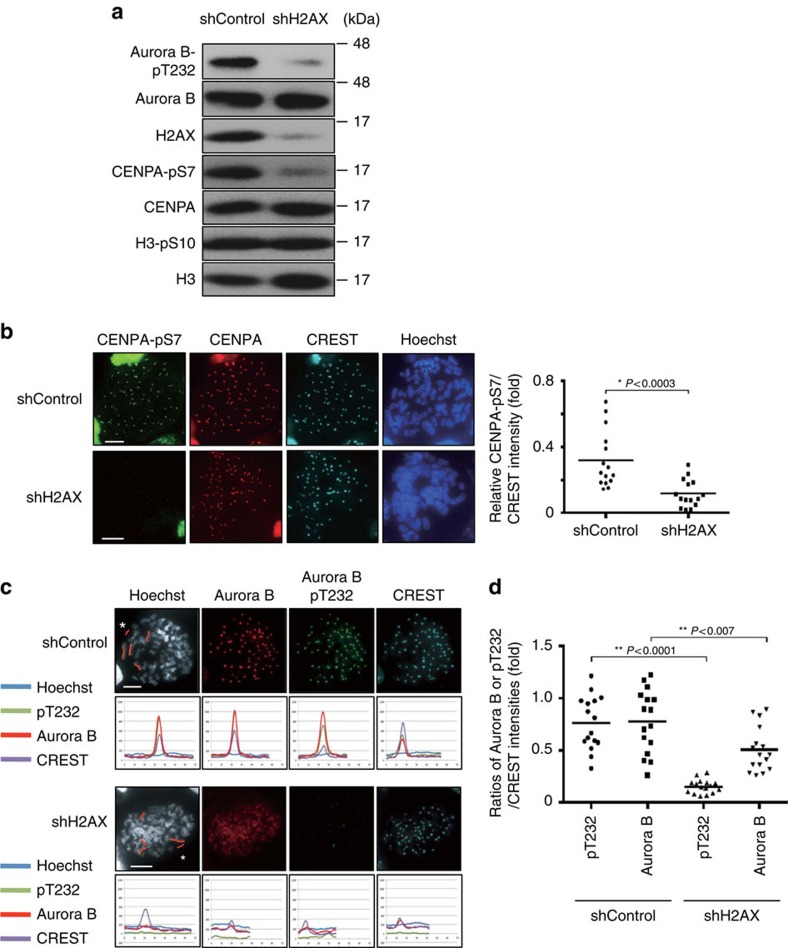
Aurora B-mediated H2AX-pS121 is a prerequisite for proper activation and deposition of Aurora B at centromeres. (**a**) HeLa cells expressing the Dox-inducible shControl or shH2AX were treated as in Fig. 2d. Chromatin fractions from mitotic cells were subjected to immunoblotting with the indicated antibodies. (**b**) Metaphase spreads from HeLa cells expressing shControl or shH2AX in the presence of doxycycline (1 μg ml^−1^) were fixed and stained with antibodies against CENPA-pS7 (green), CENPA (red) and CREST (aqua). DNA was counterstained with Hoechst (blue). Representative images are shown (left). Scale bars, 10 μm. Signal intensities of CENPA-pS7 at the centromere were measured (right) and CREST was used as a control of the centromeric signal intensity (*n*=15 cells, 5 kinetochores were measured per each cell, Student's *t*-test). (**c**) Representative images of metaphase spreads from HeLa cells expressing shControl or shH2AX cells in the presence of doxycycline (1 μg ml^−1^) (upper and third panels). Fluorescence intensities scanned along with each chromosome are indicated by red lines (second and bottom panels). Red lines marked with asterisks indicate negative backgrounds (upper and third panels). Scale bars, 10 μm. (**d**) Mitotic spreads from HeLa cells expressing the shControl or shH2AX were performed and signal intensities of AuroraB and AuroraB pT232 at the centromere were measured. CREST was used as a control of the centromeric signal intensity (*n*=16 cells, 5 kinetochores were measured per each cell, Student's *t*-test).

We then determined the physiological importance of H2AX-pS121 in Aurora B deposition and activation at centromeres. Although H2AX depletion did not affect the complex formation of CPC (Supplementary Fig. 6), it strongly suppressed Aurora B activation in early mitosis when evaluated by means of Aurora B-pT232 and CENPA-pS7 (Fig. 3a,b and Supplementary Fig. 7a). Similar impairment of Aurora B activation and deposition was also observed in H2AX KO HeLa cells (Supplementary Fig. 7b). H2AX depletion did not inhibit mitotic entry, because H3-pS10 was readily detectable in H2AX-depleted cells. This H3-pS10 was probably mediated by partially activated Aurora B by INCENP binding and its phosphorylation on the chromosome arms. Chromosome spread analysis demonstrated that H2AX depletion resulted in a reduction in Aurora B and more markedly its active phosphorylation at centromeres, and an increase in Aurora B on chromosome arms (Fig. 3c). Reduction in deposition and activation of Aurora B at centromeres were further confirmed by measuring at least 15 H2AX-depleted cells (Fig. 3d). These phenotypes in H2AX-depleted cells were very similar to those observed in Haspin-depleted cells.

### H2AX-pS121 functionally interacts with Bub1 and Haspin

**Figure 4:**
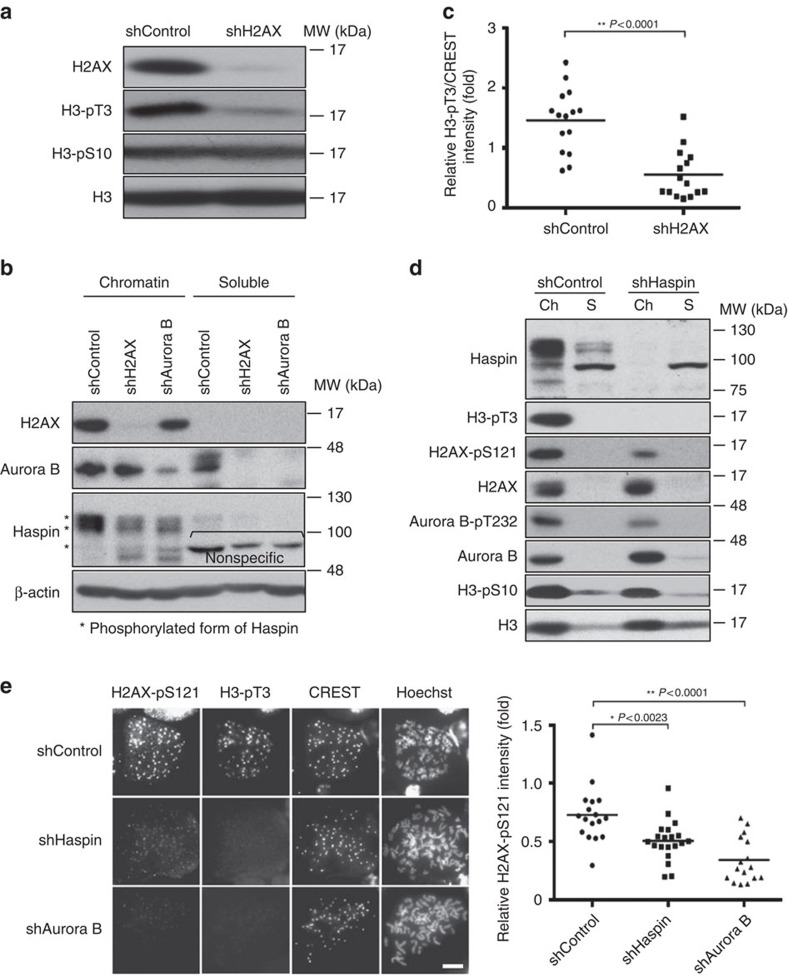
H2AX-pS121 functions upstream of Haspin-H3-pT3. (**a**) Chromatin fractions as in Fig. 2d were subjected to immunoblotting using the indicated antibodies. (**b**) HeLa cells expressing the Dox-inducible shControl, shH2AX or shAurora B were treated as in Fig. 2d. After mitotic shake-off, chromatin (Ch) and soluble (S) fractions from mitotic cells were subjected to immunoblotting using the indicated antibodies. (**c**) Chromosome spreads from HeLa cells as in Fig. 3b were stained with antibodies against H2AX, H3-pT3 and CREST (see Supplementary Fig. 8c). Signal intensities of H3-pT3 at the centromere were measured and CREST was used as a control of the centromeric signal intensity (*n*=15 cells, 5 kinetochores were measured per each cell, Student's *t*-test). (**d**) HeLa cells expressing the Dox-inducible shControl and shHaspin were treated as in Fig. 2d. After mitotic shake-off, chromatin (Ch) and soluble (S) fractions from mitotic cells were subjected to immunoblotting using the indicated antibodies. (**e**) Chromosome spreads from HeLa cells expressing the shControl, shHaspin or shAuroraB were stained with antibodies against H2AX-pS121, H3-pT3 and CREST. DNA was counterstained with Hoechst. Representative images are shown (left). Scale bars, 10 μm. Signal intensities of H2AX-pS121 at the centromere were measured (right) and CREST was used as a control of the centromeric signal intensity (*n*⩾15 cells, 5 kinetochores were measured per each cell, Student's *t*-test).

**Figure 5:**
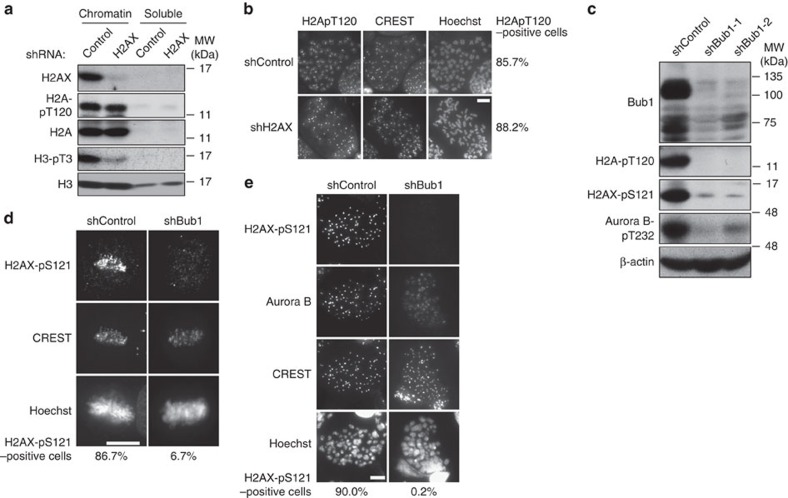
H2AX-pS121 functions downstream of Bub1-H2A-pT120. (**a**) HeLa cells expressing the Dox-inducible shControl or shH2AX were treated as in Fig. 2d. Chromatin fractions from mitotic cells were subjected to immunoblotting with the indicated antibodies. (**b**) Metaphase spreads from HeLa cells expressing shControl or shH2AX in the presence of doxycycline (1 μg ml^−1^) were fixed and stained with antibodies against H2A-pT120, CREST and Hoechst. Representative images and the percentage of the H2A-pT120-positive cells are shown (*n*⩾65). Scale bars, 10 μm. (**c**) HeLa cells expressing the Dox-inducible shControl, shBub1-1 or shBub1-2 were treated as in Fig. 2d. Chromatin fractions from mitotic cells were subjected to immunoblotting with the indicated antibodies. (**d**,**e**) HeLa cells expressing shControl or shBub1-1 were fixed for immunofluorescence (**d**) (*n*⩾25) as in Fig. 2c or for mitotic spread (**e**) (*n*⩾20) as in (**b**). Representative images and the percentage of the H2AX-pS121-positive cells are shown. Scale bars, 10 μm.

Two distinct histone marks, Haspin-mediated H3-pT3 and Bub1-mediated H2A-pT120, have been recently reported to independently regulate deposition of CPC at centromeres^15^. In addition, given that positive feedback between Haspin-H3-pT3 and Aurora B promotes CPC accumulation at centromeres^18^, we first examined whether H2AX-pS121 regulates this feedback. Immunoblotting analysis revealed that H2AX depletion and KO (Supplementary Fig. 10a), similar to Aurora B depletion (Supplementary Fig. 8a), severely compromised Haspin-mediated H3-pT3 (Fig. 4a). H2AX depletion also resulted in a downward mobility shift of Haspin bands (Fig. 4b) due to the dephosphorylation, because a similar downward shift of Haspin band was observed when mitotic chromatin fraction was treated with CIP (calf intestinal phosphatase)^18^ (Supplementary Fig. 8b). These results indicate the indispensable role of H2AX and of Aurora B in full activation of Haspin. Chromosomal spread analysis also showed that loss of H2AX dramatically decreased the signal intensity of H3-pT3 (Fig. 4c and Supplementary Fig. 8c). As expected from the fact that Haspin KD causes Aurora B displacement on centromeres, Haspin depletion resulted in a slight reduction in Aurora B-mediated H2AX-pS121 and Aurora B-pT232 (Fig. 4d). A reduction in centromeric signals of H2AX-pS121 was also confirmed in chromosome spreads, although the reduction was less effective when compared with Aurora B depletion (Fig. 4e). We then examined the interconnection between Aurora B-mediated H2AX-pS121 and Bub1-mediated H2A-pT120. Very importantly, although H2AX depletion did not affect Bub1-mediated H2A-pT120, Bub1 depletion almost completely abolished H2AX-pS121. (Fig. 5a–e). These results suggest that Bub1-H2A-pT120 probably functions upstream of Aurora B-H2AX-pS121, as Bub1 failed to phosphorylate H2AX (Supplementary Fig. 9). Taken together, Aurora B-mediated H2AX-pS121 probably creates Aurora B autoactivation circuitry at centromeres that is epistatic to Haspin-H3-pT3, but functions downstream of Bub1-H2ApT120.

### H2AX-pS121 is required for proper chromosome segregation

**Figure 6:**
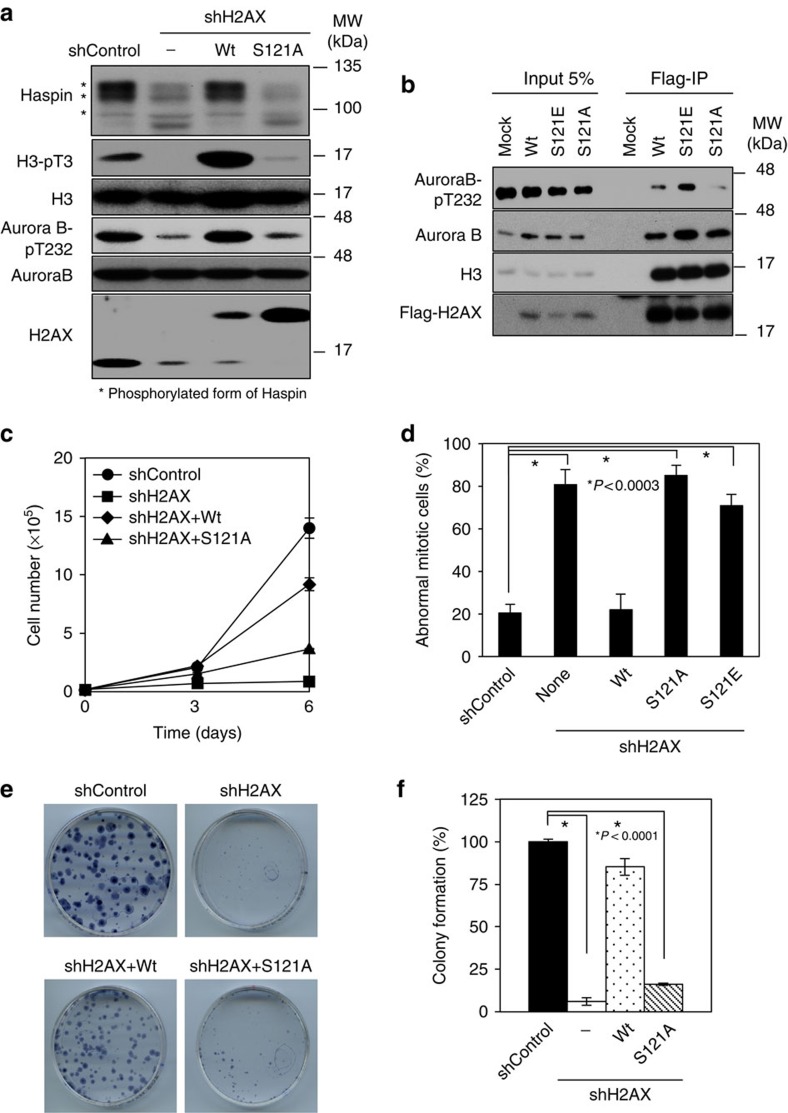
H2AX-pS121 functions as a platform for the recruitment of activated Aurora B and is essential for cell proliferation. (**a**) HeLa cells expressing the Dox-inducible shControl, shH2AX with/without Flag-tagged wild-type (Wt) or S121A mutant H2AX were treated as in Fig. 2d. Chromatin fractions were subjected to immunoblotting using the indicated antibodies. (**b**) A Flag pull-down assay was performed using lysates from nocodazole-treated 293T cells expressing Flag-H2AX WT, S121E or S121A. Bound proteins were subjected to immunoblotting using the indicated antibodies. (**c**) HeLa cells as in **a** were cultured in the presence of doxycycline (1 μg ml^−1^) and their numbers were counted at the indicated times. Data are shown as means ±s.d. of at least three independent experiments. (**d**) HeLa cells with H2B-EGFP expressing the Dox-inducible shControl, shH2AX and Flag-H2AX-WT, H2AX-S121A or H2AX-S121E were subjected to time-lapse imaging and mitotic progression was observed. Quantification of cells showing abnormal mitosis is shown. Data are expressed as means±s.d. of at least three independent experiments (*n*⩾20, Student's *t*-test). (**e**,**f**) A quantitative colony formation assay was performed using cells as in **c** and data are shown as means ±s.d. of at least three independent experiments (Student's *t*-test).

We then determined whether the mitotic phenotypes observed in H2AX-depleted cells were due to the impaired Aurora B-mediated phosphorylation at S121. Introduction of wild-type H2AX, but not its S121A mutant, in H2AX-depleted cells effectively rescued Aurora B activation, Haspin phosphorylation and the level of H3-pT3 (Fig. 6a). Thus, the results clearly indicate that Aurora B-mediated H2AX-pS121 plays a critical role in the culminated activation of Aurora B at centromeres, triggering activation of the Haspin-H3-pT3 pathway. We hypothesized that Aurora B-mediated H2AX-pS121 could provide a molecular scaffold for Aurora B autoactivation circuitry and activation of Haspin-H3-pT3. To examine this possibility, we analysed protein complexes that bound to H2AX-pS121. Very intriguingly, ectopic expression of a phosphomimic mutant of S121E, a phosphorylation site mutant of S121A and wild-type H2AX in HeLa cells revealed that S121E bound more Aurora B and activated Aurora B (pT232) than did the wild-type and S121A (Fig. 6b). Wild-type H2AX and these mutants bound equally to histone H3 as a control. Thus, the results propose a model in which H2AX-pS121 likely functions as a platform for Aurora B auto-activation circuitry, leading to activation of Haspin-H3-pT3.

Finally, we asked whether the defective cell proliferation and abnormal mitoses of H2AX-depleted cells was due to Aurora B-mediated H2AX-pS121. Introduction of wild-type H2AX to H2AX-KD and H2AX-KO HeLa cells effectively recovered defective cell proliferation and abnormal mitoses, whereas the S121A mutant did not (Fig. 6c,d and Supplementary Fig. 10a–c). A colony formation assay also demonstrated a similar impairment in the ability of the S121A mutant to overcome the defects in cell proliferation in H2AX-depleted cells (Fig. 6e,f). Unexpectedly, an add-back of S121E also failed to rescue the mitotic abnormalities found in H2AX-KD HeLa cells (Fig. 6d). This might be explained by the observation that ectopically expressed S121E failed to predominantly localize to centromeres.

### Haspin KO mice are apparently normal and fertile

**Figure 7:**
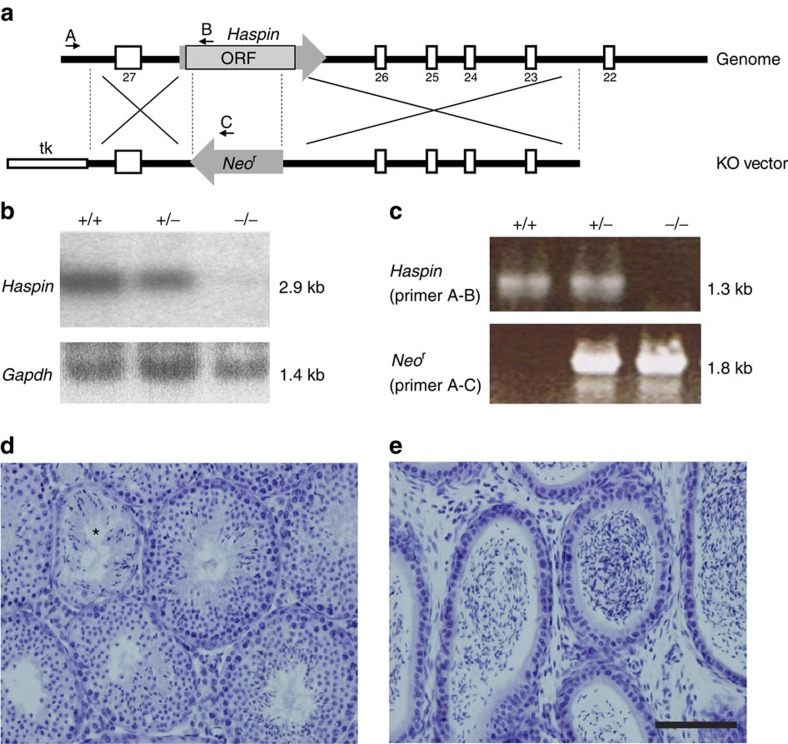
Haspin KO mice are viable but show testicular abnormalities. (**a**) Schematic representation of the methods used for gene targeting of the Haspin genome. The gene-targeting construct contained Neor (grey arrow). Open boxes indicate exons of intergrin alphaE and TK. Black arrows indicate the primers used in the PCR for determining genotypes. (**b**) Northern blotting for Haspin expression. Haspin transcripts were not detected in testes from Haspin^−/−^ mice. Gapdh cDNA was used as an internal control. Nucleotide sizes are indicated on the right. (**c**) Genotyping of mice using PCR: +/+ (wild-type), +/− (heterozygous mutant), −/− (homozygous mutant). (**d**) Representative haematoxylin- and eosin-stained testicular sections of Haspin^−/−^ mice. The asterisk indicates a tubule with disordered germ cells. (**e**) Hematoxylin- and eosin-stained epididymis of Haspin^−/−^ mice. Scale bar, 100 μm.

Remarkably, H2AX KO mice are viable and developmentally normal, although they are growth retarded, immune-deficient and infertile^29^. This suggests that there might be a parallel pathway(s) that bypasses activation of Haspin-H3-pT3 in the absence of H2AX *in vivo*. To examine this possibility, we generated Haspin KO mice to compare their phenotypes with those of the H2AX KO mice and found that the former mice were also viable and exhibited no remarkable phenotypic change in body weight or organs, although some germ cells were disordered in a portion of Haspin-deleted testes (Fig. 7a–e). These results suggest that viable and developmentally normal phenotypes in H2AX KO mice are not likely to be due to bypassed activation of Haspin-H3-pT3 by unidentified parallel pathways other than Aurora B-H2AX-pS121.

## Discussion

**Figure 8:**
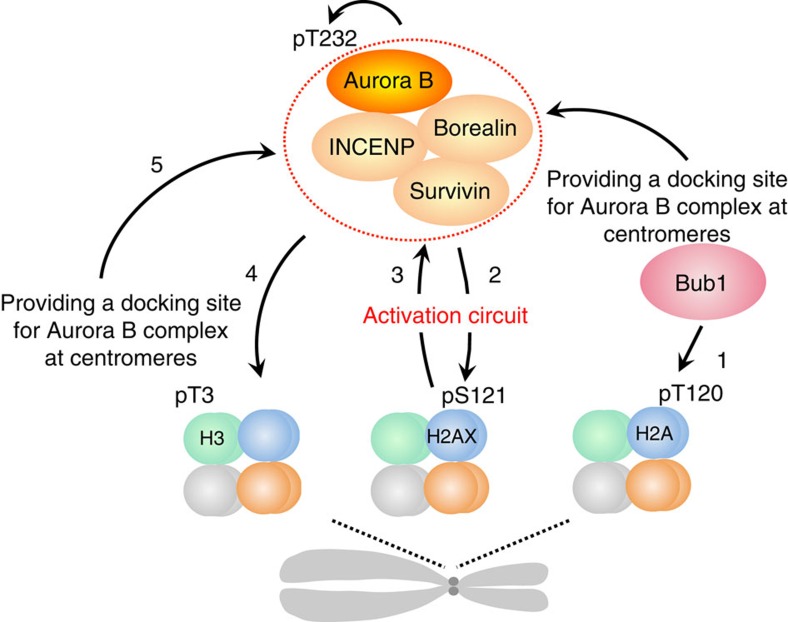
Schematical model of Aurora B autoactivation circuitry. Bub1 phosphorylates centromeric H2A at T120 (1), which induces accumulation of CPC complex to centromeres. Partially activated Aurora B phosphorylates H2AX at S121 (2) leading to abrupt and culminated activation of Aurora B at inner centromeres (3), which in turn activates Haspin (4) and regulates further deposition of Aurora B at the inner centromere (5).

Our present results clearly revealed the Aurora B-mediated H2AX-pS121 plays an important role in full activation of Aurora B at centromeres in early mitosis, which in turn epistatically regulates the Haspin-H3-pT3 pathway (Fig. 8). Autophosphorylation in *trans* probably forms one of the most simple positive or negative feedback loops *in vivo* and the outcome generally depends on the kinase concentration. In our case, Aurora B-mediated H2AX-pS121 provided a platform for activated Aurora B at the centromere, increasing the concentration of activated Aurora B in this region. This autoactivation circuitry in addition to Haspin-pT3 probably supplies sufficient Aurora B activity at inner centromeres and generates a strong gradient of activity along the two chromatid axes. Aurora B-dependent phosphorylation of outer kinetochore proteins promotes destabilization of kinetochore-microtubule attachment. Thus, the gradient of its kinase activity probably links tension-dependent stretching of sister kinetochores with stabilization of proper attachment during chromosome alignment.

Activation and recruitment of Aurora B is regulated by multiple mechanisms. The Bub1-H2ApT120-Shugoshin pathway promotes Aurora B recruitment to the centromere through targeting of Borealin, a CPC component^34^, and regulates Haspin-H3-pT3 pathway^18^. This idea is consistent with our results, as H2AX-pS121 was almost completely dependent on Bub1. pT120-Shugoshin-dependent recruitment of Aurora B to the centromere could be a first trigger in initiation of Aurora B-H2AX-pS121 autoactivation circuitry.

Although, similar to H2AX KO mice, Haspin KO mice were apparently normal and fertile, Haspin inhibition did result in compromised metaphase chromosome alignment and spindle checkpoint signalling^35^. It is not yet clear how the phosphorylation of Haspin by Aurora B contributes its function; however, it is possible that H2AX-pS121 and Haspin-H3-pT3 act redundantly in culminated and circumscribed activation of Aurora B at centromeres. Thus, it should be of great interest to examine the phenotypes in H2AX and Haspin double KO mice.

## Methods

### Cell culture and drug treatment

HeLa (ATCC) cells and MEFs (gift by Dr S. Ellegde) were cultured in DMEM medium supplemented with 10% fetal bovine serum and 1% penicillin–streptomycin (Invitrogen). All cells were cultured at 37 °C under 5% CO_2_.

### Antibodies

Polyclonal antibodies specific for a phosphorylated form of H2AX at S121 were generated in rabbits with the keyhole limpet haemocyanin-conjugated peptide LPKKT(pS)ATVGP as an antigen. Antibodies used in this study are listed in Supplementary Table 1.

### Plasmid construction

To construct Tet-on-inducible lentivirus constructs, complementary DNA of human H2AX and Bub1 was inserted into a pENTR1A vector (Invitrogen) containing 3 × Flag epitope. To construct H2B-EGFP lentivirus vectors, cDNA of H2B was inserted into a pENTR1A vector containing enhanced grren fluorescent protein (EGFP). S121A and S121E mutations of pENTR1A 3 × Flag H2AX were generated by inverse PCR with a Site-Specific Mutagenesis Kit (Toyobo). EGFP-tagged H2AX expression vectors were constructed in a previous study^36^. The resultant plasmids were mixed with CSIV-TRE-RfA-UbC-Puro vector or CSII-CMV-MCS-IRES2-Bsd vector (a gift of H. Miyoshi) and treated with Gateway LR clonase to generate the lentivirus vectors.

### CRISPR/Cas9-mediated gene KO

A sgRNA for human H2AX was ordered as oligonucleotides, annealed and cloned into the dual Cas9 and sgRNA expression vector pX330 (kindly provided by Dr Feng Zhang) with a BbsI site. The plasmid (pX330-hH2AX-1) was transfected into HeLa cells using Lipofectamine2000 (Invitrogen) according to the manufacturer's protocol. After 48 h incubation, the cells were split individually to make a clonal cell line. The ∼500 bp genomic fragments containing the target in the centre were PCR amplified and sequenced to confirm the gene disruption. SgRNA sequence: human H2AX-1 (5′- CGCCAACGCGCTCGGCGTAG -3′).

### Lentiviruses

Lentiviruses expressing the respective shRNAs and genes were generated as described previously^37^. GFP-H2B-expressing cells were described previously^38^. Cells infected with viruses were treated with 10 μg ml^−1^ blasticidin for 2 days with or without 2 μg ml^−1^ puromycin (Sigma-Aldrich). All the target sequences for lentivirus-based shRNAs are summarized in Supplementary Table 2.

### Immunoblotting

For preparation of total cell lysates, collected cells were washed, lysed directly with sample buffer (2% SDS, 10% glycerol, 100 mM dithiothreitol (DTT), 0.1% bromophenol blue, 50 mM Tris-HCl at pH 6.8) and boiled for 5 min. Chromatin fractionation was performed as follows. In brief, 5 × 10^5^ cells were suspended in 200 μl of solution A (10 mM HEPES pH 7.9, 10 mM KCl, 1.5 mM MgCl_2_, 0.34 M sucrose, 10% glycerol, 1 mM DTT, protease and phosphatase inhibitors and 0.1% Triton X-100). The cells were incubated on ice for 5 min and cytoplasmic fractions were harvested by centrifugation at 1,300 *g* for 4 min. The isolated nuclei were washed in solution A, lysed in 100 μl solution B (3 mM EDTA, 0.2 mM EGTA, 1 mM DTT, protease and phosphatase inhibitors) and incubated on ice for 10 min. The soluble nuclear fractions were harvested by centrifugation at 1,700 *g* for 4 min. Insoluble chromatin was then washed in solution B and resuspended in sample buffer. Proteins (5–50 μg) were separated by SDS–PAGE, transferred to a polyvinylidene difluoride membrane (Immobilon-P; Millipore) and then subjected to immunoblotting^39^. Full scans of the most important blots are provided in the Supplementary Fig. 11.

### Cell cycle synchronization

To synchronize cells at the G1/S boundary, cells were treated with 2 mM thymidine for 18 h and then released into S phase by washout thymidine with PBS and the addition of medium. After 8 h release, these cells were exposed to 2 mM thymidine for 18 h and released again^36^. To synchronize cells at the G2 phase, cells were treated with 4.5 μM RO-3306 for 28 h and released. To synchronize cells at prometaphase, cells were treated with 0.1 μg ml^−1^ nocodazole for 12–18 h and collected by shake-off. To monitor the cell cycle profile, cells were fixed with 70% ethanol at the indicated times and DNA was counterstained with 0.05 mg ml^−1^ propidium iodide containing RNase for 30 min at 37 °C. Flow cytometry was performed using a FACSVerse (BD Biosciences).

### *In vitro* kinase assay

Recombinant kinase-active Aurora B (Sigma, A2108) was incubated with 1 μg protein substrate in 25 μl kinase buffer (50 mM Tris-HCl pH 7.5, 10 mM MgCl_2_, 10 mM MnCl_2_, 1 mM DTT and 10 μM ATP) in the presence or absence of ZM-447439 (Merck) for 30 min at 30 °C. Substrate phosphorylation was analysed by SDS–PAGE and was monitored using H2AX-pS121 antibodies.

### Immunoprecipitation

Chromatin from mitotic HeLa cells was prepared and solubilized with IP kinase buffer (50 mM HEPES pH 8.0, 150 mM NaCl, 2.5 mM EGTA, 1 mM DTT, 0.1% Tween 20 and 10% glycerol) containing a cocktail of protease inhibitors for 10 min on ice and sonicated. Next, 400 μg protein were immunoprecipitated with 1 μg Borealin antibodies for 12 h at 4 °C, followed by incubation with protein G-agarose (GE Healthcare) for 1 h. Full scans of the most important blots are provided in the Supplementary Fig. 11.

### Calf intestinal phosphatase assay

Mitotic chromatin was prepared and incubated with/without ten units CIP (New England Biosciences) in NEB buffer 3 (50 mM Tris-HCl pH 8.0, 100 mM NaCl, 10 mM MgCl_2_ and 1 mM DTT) at 37 °C.

### Time-lapse microscopy

Cells were cultured in 24-well plates for 24 h in the presence of doxycycline. Images were acquired using an In Cell Analyzer 2000 (GE Healthcare) at 37 °C and 5% CO_2_ in the Core Laboratory of Nagoya City University. Time-lapse images were captured every 10 min with a 10 μm *z* axis projection, using × 60/0.95 Plan Apo and × 20/0.45 Plan Fluor objectives (Nikon). All images were analysed with Image J software.

### Immunofluorescence

Cells on cover slips were fixed in ice-cold methanol/acetone for 10 min at −20 °C, permeabilized with 0.5% TritonX-100 in PBS for 10 min and incubated in blocking buffer (5% goat serum and 0.3% Tween 20 in PBS) for 30 min. Samples were then incubated with anti-H2AX-pS121 and CREST diluted in blocking buffer for 1 h at room temperature, followed by incubation with anti-rabbit IgG conjugated with Alexa Fluor 488 (Life Technologies) and anti-human IgG conjugated with Alexa Fluor 594 (Life Technologies) secondary antibodies diluted in blocking buffer (1:200) for 1 h at room temperature. Nuclei were counterstained with Hoechst 33342 (1:1,000). Images were acquired with 0.2 μm sections using a DeltaVision Elite (Applied Precision) comprising an Olympus IX71 wide-field inverted fluorescence microscope, with a × 60/1.42 PlanApo N objective (Olympus), an oil-immersion objective and a Photometrics CoolSnap HQ2 camera (Roper Scientific). Images were maximum intensity projections of deconvolved stacks obtained using SoftWoRx software (Applied Precision).

### Chromosome spreads

Cells were treated with nocodazole (0.2 μg ml^−1^) for 60 min and mitotic cells were collected by shake-off. Cells were suspended in hypotonic buffer (75 mM KCl:0.8% NaCitrate:H_2_O at 1:1:1) with 150 nM okadaic acid and protease inhibitors at 3 × 10^5^ cells per ml. Next, 200 μl of the swollen cell suspension were spun in a cytospin at 1,800 r.p.m. for 10 min (Fig. 2e) or at 1,500 r.p.m. for 5 min (in other experiments). Slides were then immersed in KCM buffer (120 mM KCl, 20 mM NaCl, 10 mM Tris/HCl pH 8.0, 0.5 mM EDTA and 0.1% Triton X-100) for 10 min (Fig. 2e). Staining with anti-H2AX-pS121 and Aurora B antibodies was carried out in KCM containing 1% BSA for 1 h at 4 °C. Slides were washed twice in KCM buffer, incubated with secondary antibodies in KCM containing 1% BSA and fixed with methanol/acetic acid (3:1) twice for 5 min each time. Next, slides were treated with Hoechst and rinsed with PBS. The slides were fixed with cold 80% ethanol in PBS for 30 min at −20 °C, followed by treatment with cold acetone and were then washed with PBS (Fig. 3b). Staining with CENPA, CENPA-pS7 and CREST antibodies was carried out in dilution buffer (10% fetal bovine serum, 0.1% Triton X-100, 120 mM KCl, 20 mM MgCl_2_, 0.5 mM EDTA and 10 mM Tris/HCl pH 8.0) for 16 h at 4 °C. In other experiments, slides were fixed with 2% paraformaldehyde (PFA) in PBS for 20 min and washed with PBS. Staining with the antibodies was carried out as in Fig. 3b.

### Quantitative immunofluorescence

Images were obtained at identical illumination settings and quantification of immunofluorescence was performed essentially as described^40^ using ImageJ software. In brief, pixel intensities along chromosomes (Fig. 3c) or of at least 50 kinetochore pairs from 10 cells (Figs 3b,d and 4c,e) were measured and background pixel intensities were subtracted. Measurements were normalized against CREST pixel values.

### Flag pull-down assay

293T cells expressing 3 × Flag H2AX WT, S121E and S121A were treated with nocozadole for 14 h. Chromatin fractions were prepared as described previously^15^ and were incubated with anti-Flag beads (M2 agarose, Sigma) for 2 h at 4 °C. The beads were washed and boiled in the SDS sample buffer and the proteins were detected by SDS–PAGE and immunoblotting. Full scans of the most important blots are provided in the Supplementary Fig. 11.

### Colony formation assay

Cells (3.3 × 10^2^) were plated in 60-mm dishes and incubated for 2 weeks. Colonies were fixed with methanol/acedic acid (1:1) for 15 min, stained with 0.4% Trypan blue (Sigma) in 20% ethanol in PBS for 15 min and counted.

### Generation of Haspin KO mice

The Haspin-deleted mouse was generated by conventional homologous recombination in embryonic stem cells. All mice retained a C57Bl/6 background. The distribution of genotypes was found to be consistent with the expected Mendelian rule of 1:2:1 on confirming the genotypes of offspring generated by crossing heterozygotes. The primer sets used for determining the genotypes were as follows. Primer A, 5′- TGCTAGGAGCCGAAGCAACATATCC -3′; Primer B, 5′- GTACGTTCGAAAGAGCCGGGTACC -3′; Primer C, 5′- CTTGACGAGTTCTTCTGAGG -3′. No transcription of messenger RNA was present in Haspin^−/−^ mice. All experimental procedures conformed to the Regulations for Animal Experimentation at Research Institute for Microbial Diseases, Osaka University, reviewed by the Institutional Laboratory Animal Care and Use Committee of Osaka University and finally approved by the provost.

### Data availability

The authors declare that the data supporting the findings of this study are available within the article. The data that support the findings of this study are available from the corresponding author upon request.

## Additional information

**How to cite this article:** Shimada, M. *et al*. Essential role of autoactivation circuitry on Aurora B-mediated H2AX-pS121 in mitosis. *Nat. Commun.* 7:12059 doi: 10.1038/ncomms12059 (2016).

## Supplementary Material

Supplementary Information Supplementary Figures 1-11 and Supplementary Tables 1-2

Supplementary Movie 1 Time-lapse imaging of mitoses in HeLa shControl cells: HeLa cells stably expressing EGFP-H2B and the Dox-inducible shControl were cultured in the presence of doxycycline (1 μg/ml). After 24 hrs, images were acquired with IN Cell Analyzer 2000 and captured every 10 min.

Supplementary Movie 2 Representative images of misalignment in H2AX-depleted HeLa cells: HeLa cells stably expressing EGFP-H2B and the Dox-inducible shH2AX were cultured in the presence of doxycycline (1 μg/ml). After 24 hrs, images were acquired with IN Cell Analyzer 2000 and captured every 10 min.

Supplementary Movie 3 Representative images of lagging chromosome in H2AX-depleted HeLa cells: HeLa cells stably expressing EGFP-H2B and the Dox-inducible shH2AX were cultured in the presence of doxycycline (1 μg/ml). After 24 hrs, images were acquired with IN Cell Analyzer 2000 and captured every 10 min.

Supplementary Movie 4 Representative images of the defects in cytokinesis in AuroraB-depleted HeLa cells: HeLa cells stably expressing EGFP-H2B and the Dox-inducible shAuroraB were cultured in the presence of doxycycline (1 μg/ml). After 24 hrs, images were acquired with IN Cell Analyzer 2000 and captured every 10 min

Supplementary Movie 5 Representative images of cell death during mitosis in AuroraB-depleted HeLa cells: HeLa cells stably expressing EGFP-H2B and the Dox-inducible shAuroraB were cultured in the presence of doxycycline (1 μg/ml). After 24 hrs, images were acquired with IN Cell Analyzer 2000 and captured every 10 min.
