# Supplementary material for: Essential role of autoactivation circuitry on Aurora B-mediated H2AX-pS121 in mitosis
Source: Nat Commun. 2016 Jul 8;7:12059. doi: 10.1038/ncomms12059 (PMC4941122; doi:10.1038/ncomms12059)
Supplement: Supplementary Information — Supplementary Figures 1-11 and Supplementary Tables 1-2 [file ncomms12059-s1.pdf]

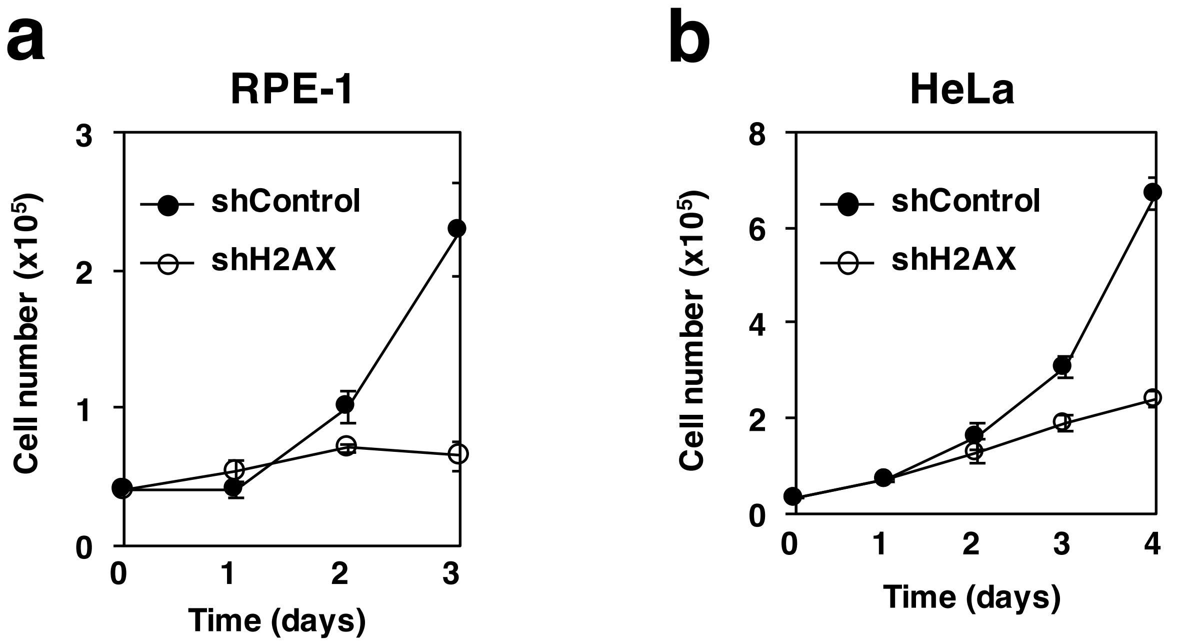

Supplementary Figure 1

Reduction of cell growth in RPE-1 and HeLa cells after H2AX knock-down.
RPE-1 cells **(a)** or HeLa cells **(b)** expressing the Dox-inducible shControl or shH2AX were cultured in the presence of doxycycline (1 μg/ml). Cell numbers were counted at the indicated times. Data are shown as means ± SD of at least three independent experiments.

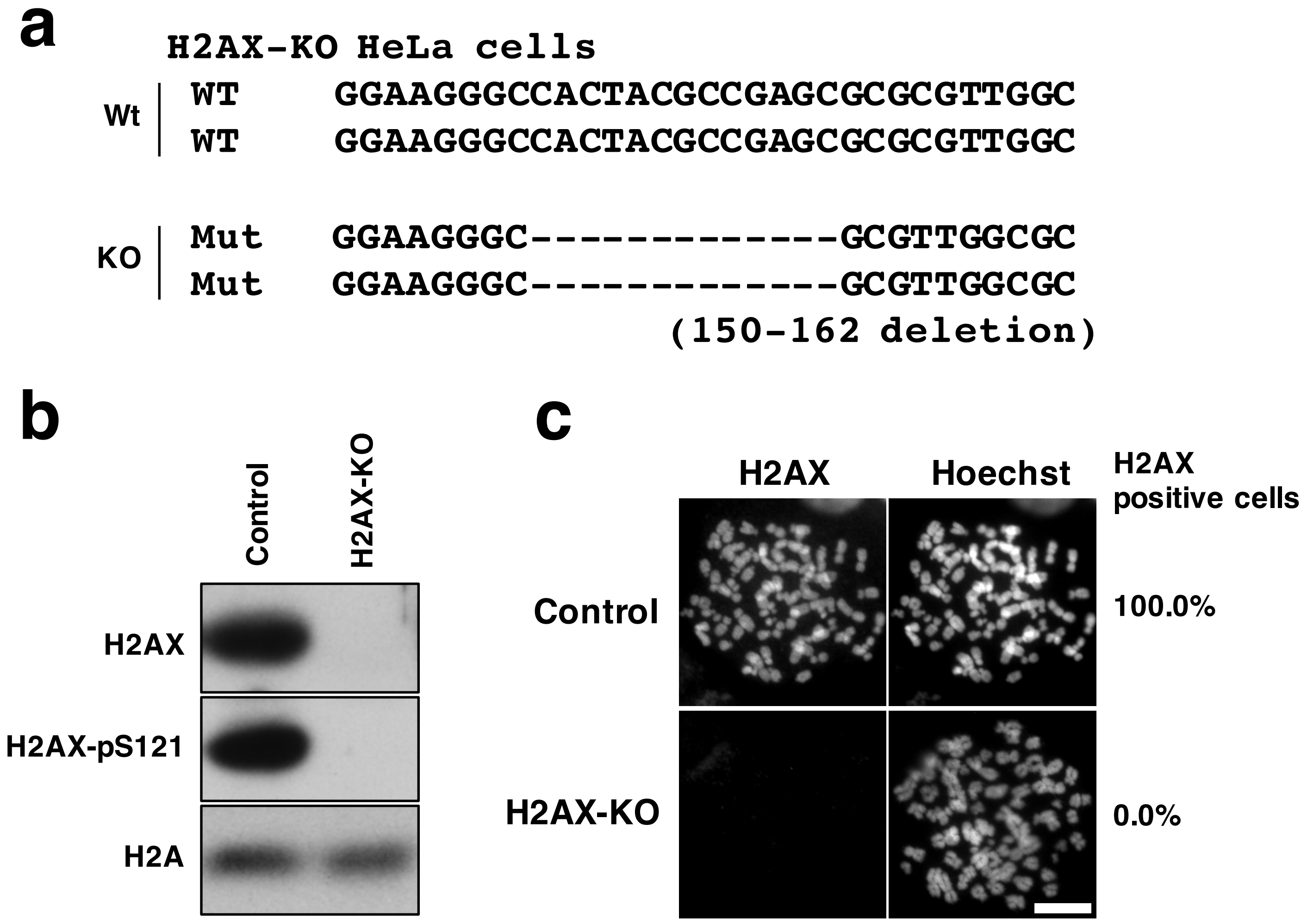

Supplementary Figure 2

Generation of H2AX-KO HeLa cells.
(a) H2AX-KO HeLa cells were generated by the use of the CRISPR/CAS9-mediated genome editing system. The wild-type H2AX gene and its mutant allele are shown. **(b)** Cell lysates from control and H2AX-KO HeLa cells were subjected to immunoblotting using the indicated antibodies. **(c)** Chromosome spreads of control and H2AX-KO HeLa cells were stained with anti-H2AX antibodies. DNA was counterstained with Hoechst. Representative images and the percentages of H2AX positive cells are shown ($n \geq 16$) . Scale bar;10μm

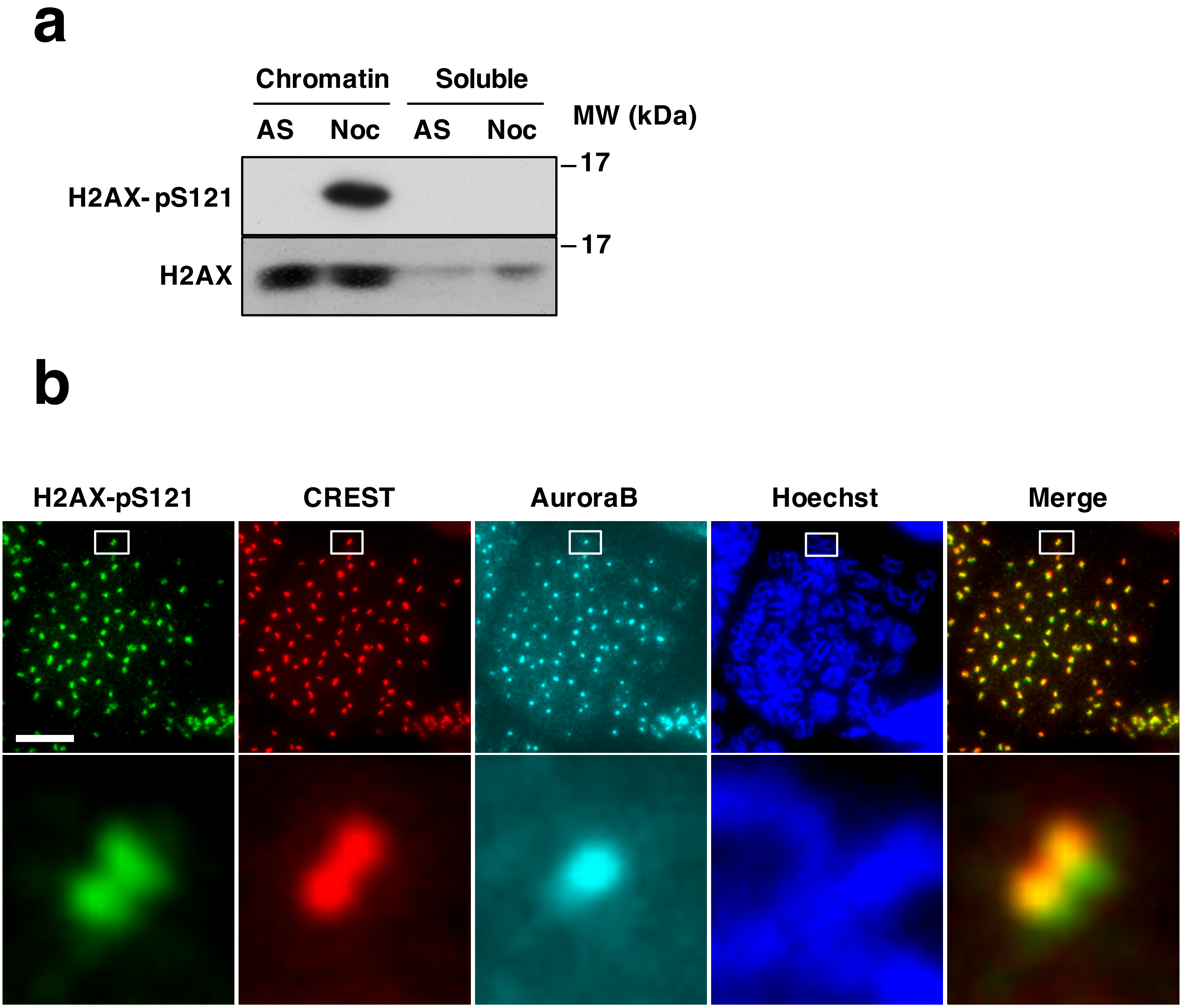

Supplementary Figure 3

H2AX-pS121 predominantly localizes at centromeres.
(a) Chromatin and soluble fractions from asynchronized (AS) and synchronized prometaphase HeLa cells obtained by shake-off after treatment with nocodazole (Noc) were subjected to immunoblotting using the indicated antibodies. **(b)** Chromosome spreads from nocodazole-treated HeLa cells were immunostained with the indicated antibodies. DNA was counterstained with Hoechst (blue). Co-localization of H2AX-pS121 and CREST at centromeres was observed (n=55). Merge: H2AX-pS121 and CREST, Scale bar;10μm

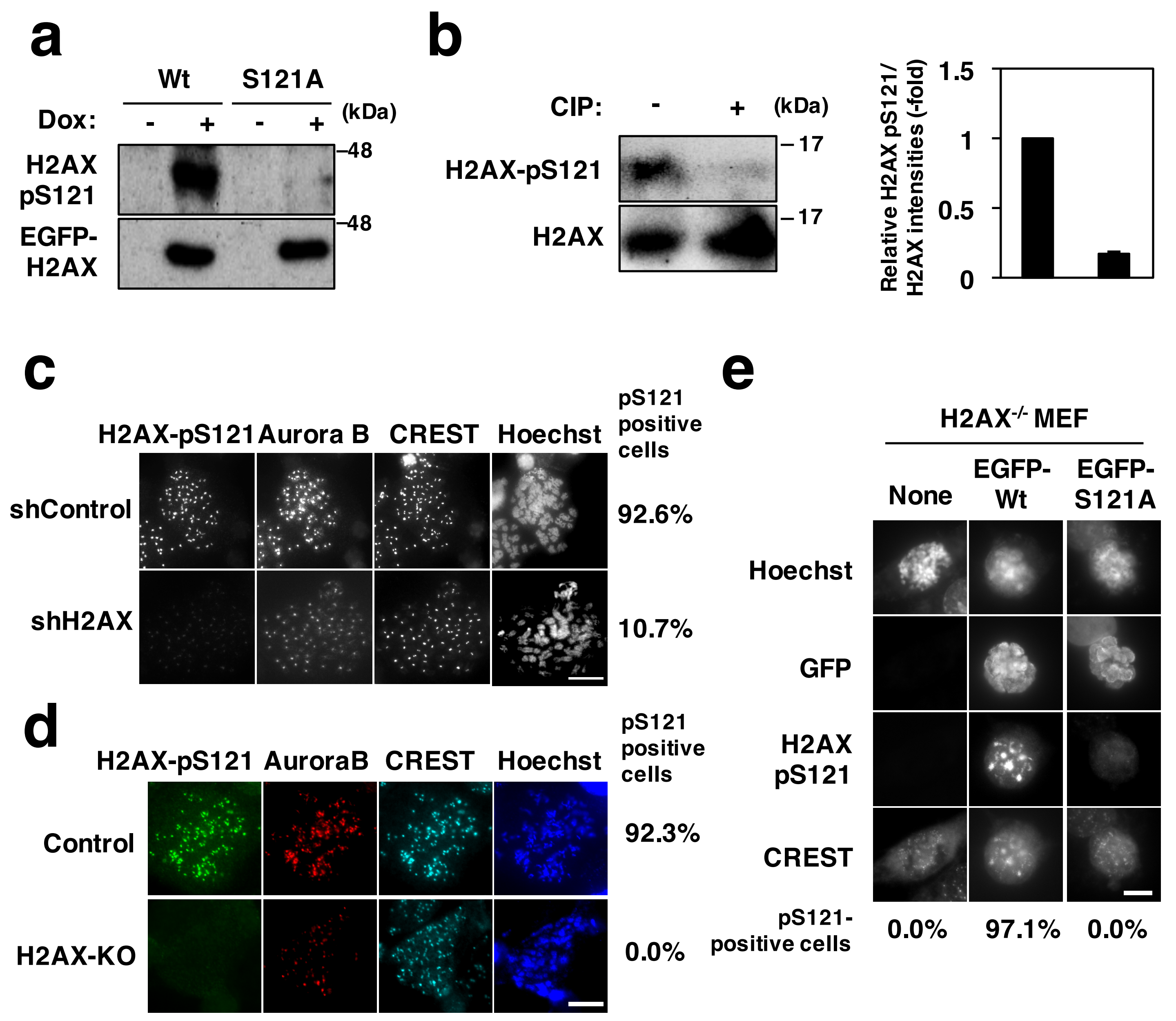

Supplementary Figure 4

Specificity of H2AX-pS121 antibodies

(a) HeLa cells expressing the Dox-inducible EGFP-tagged wild-type H2AX or its S121A mutant were cultured in the presence of doxycycline (1 μg/ml) for 2 days and treated with nocodazole (0.1 μg/ml) for 12 hrs. After mitotic shake-off, cells were subjected to immunoblotting using the indicated antibodies. **(b)** Chromatin fractions from nocodazole-treated HeLa cells were incubated with or without calf intestinal phosphatase (CIP) at 37 ° C for 2 hrs and then subjected to immunoblotting using the indicated antibodies. Intensities of each band were measured by Image J and the relative H2AX-pS121/H2AX intensities are shown in right.

(c) (d) Chromosome spreads from HeLa cells expressing shControl or shH2AX **(c)** and control and H2AX KO HeLa cells **(d)** were stained with the indicated antibodies. DNA was counterstained with Hoechst. Representative images and the percentages of H2AX-pS121 positive cells are shown (n≥18) . Scale bar;10μm **(e)** H2AX$^{-/-}$ MEF cells and those expressing EGFP-wild type H2AX or its S121A mutant were fixed and stained with the indicated antibodies. DNA was counterstained with Hoechst. Proportions of H2AX-pS121-positive cells are shown below (n≥20) . Scale bar;10μm

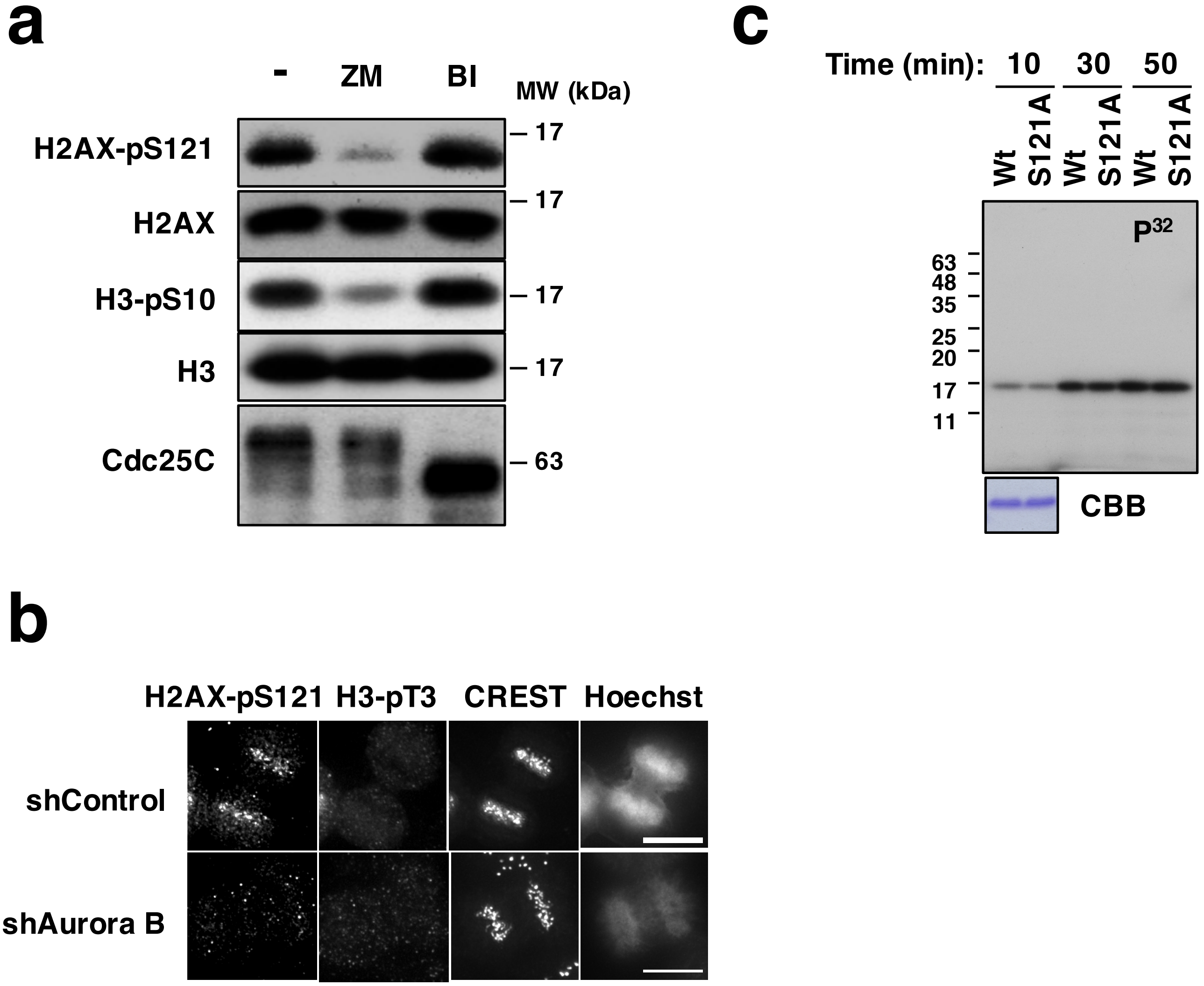

Supplementary Figure 5

Aurora B phosphorylates H2AX at S121 both *in vivo* and *in vitro*

(a) HeLa cells synchronized by nocodazole (0.1 μg/ml) were treated with or without 2 μM ZM447439 (ZM) and 4 μM BI2546 (BI) for 2 hrs or 4 hrs, respectively. Mitotic cells were collected by mitotic shake-off, and chromatin fractions were subjected to immunoblotting using the indicated antibodies. **(b)** HeLa cells expressing shControl or shAurora B were fixed and stained with the indicated antibodies. Representative images of anaphase cells are shown. Scale bar;10μm **(c)** *In vitro* kinase assay using recombinant GST-Aurora B and purified wild-type hH2AX or its S121A mutant. Kinase reaction was performed at 30° C for the indicated time.

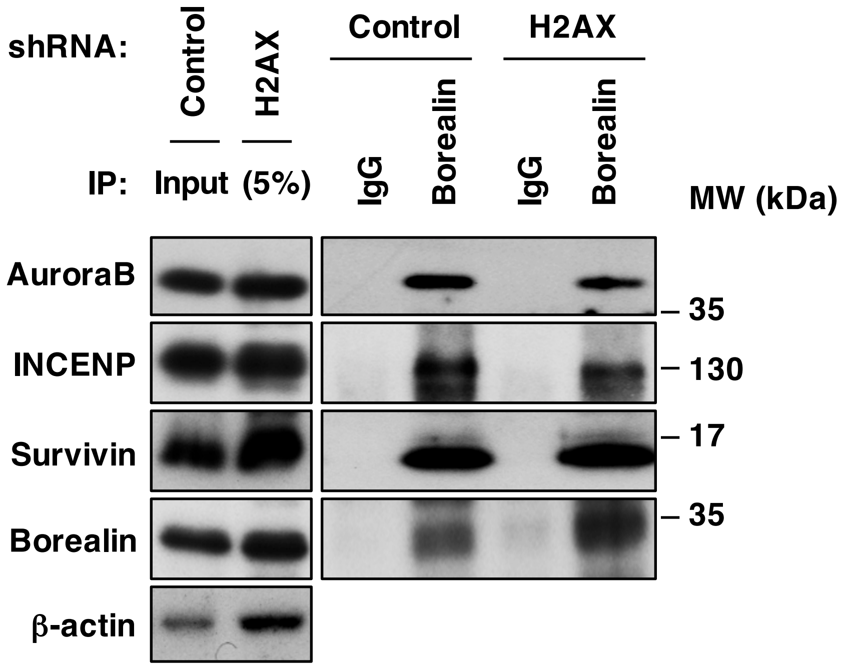

Supplementary Figure 6

Formation of CPC complex is not affected by H2AX depletion
HeLa cells expressing the Dox-inducible shControl or shH2AX were treated as in Figure 2d. Total extracts were subjected to immunoprecipitation with anti-Borealin antibody or control IgG, and the resultant precipitates as well as inputs (5%) were then analyzed by immunoblotting using the indicated antibodies.

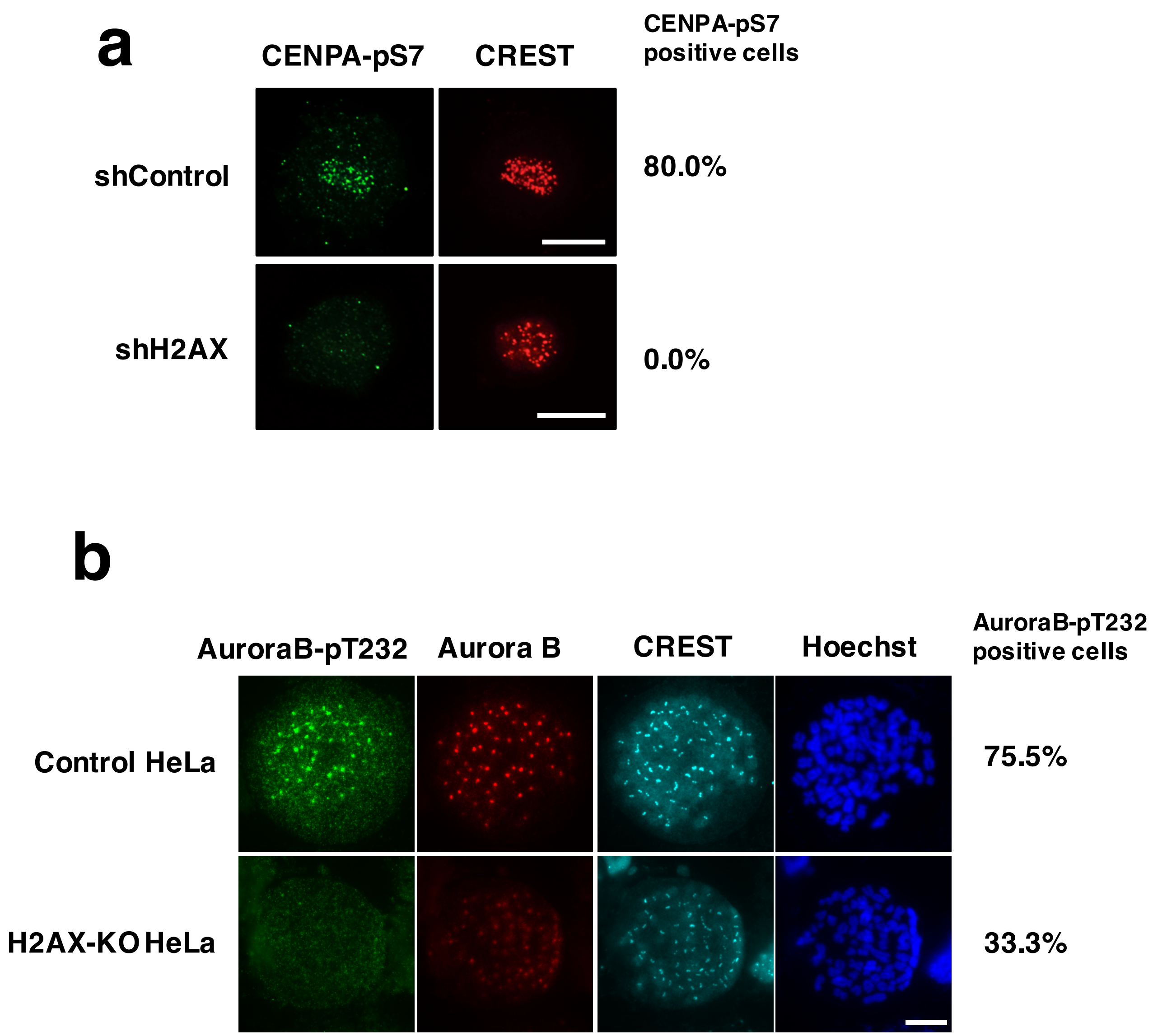

Supplementary Figure 7

CENPA-pS7, Aurora B phosphorylation and accumulation at centromeres are impaired in H2AX-KO HeLa cells.
(a) HeLa cells expressing the Dox-inducible shControl or shH2AX were incubated in the presence of RO3306 (9 μM) and doxycycline (1 μg/ml) for 24 hours, and then released into medium containing doxycycline and MG132. Two hours after release, cells were fixed with MeOH/acetone and stained with the indicated antibodies. Representative images and the percentages of CENPA-pS7 positive cells are shown (n≥10) . Scale bars, 10 μm **(b)** Chromosome spreads from H2AX-KO or control HeLa cells were stained with the indicated antibodies. DNA was counterstained with Hoechst. Representative images and the percentages of Aurora B-pT232 positive cells are shown (n≥27) . Scale bar;10μm

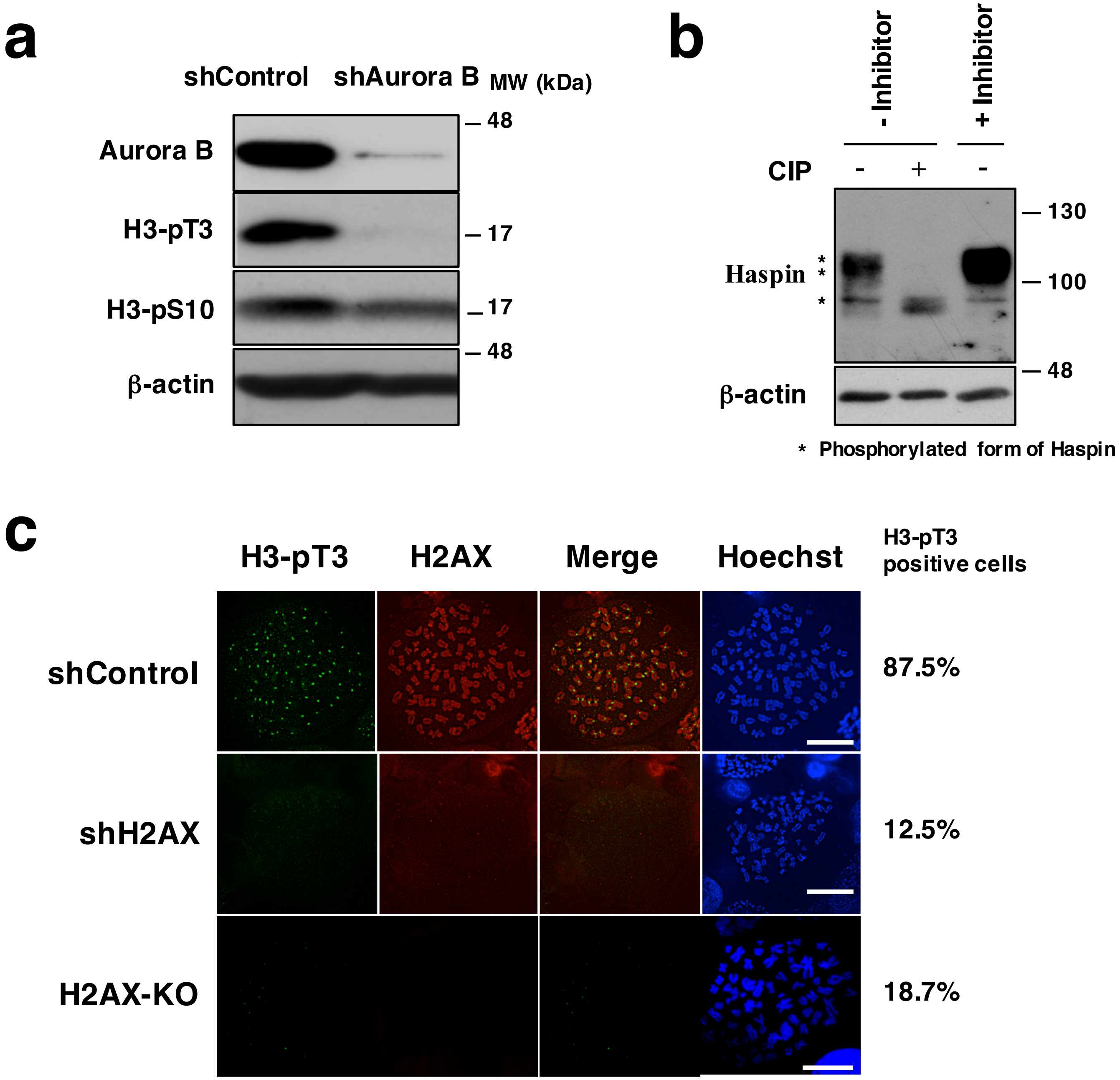

Supplementary Figure 8

Phosphorylation of H3 at T3 is strongly compromised in H2AX-depleted HeLa cells
(a) HeLa cells expressing the Dox-inducible shControl or shAuroraB were cultured in the presence of doxycycline (1 μg/ml) for 3 days and treated with nocodazole (0.1 μg/ml) for 12 hrs. After mitotic shake-off, cell extracts were subjected to immunoblotting using the indicated antibodies. **(b)** Phosphorylation of Haspin during mitosis. Haspin is hyper phosphorylated in mitosis. Mitotic chromatins prepared with or without phosphatase inhibitors were incubated with or without CIP (Calf Intestinal Alkaline Phosphatase) at 37° C for 30 min and then subjected to immunoblotting using the indicated antibodies. **(c)** Chromosome spreads from HeLa cells as in (**a**) were stained with antibodies against H3-pT3 (green), and H2AX (red). DNA was counterstained with Hoechst (blue). Representative images and the percentages of H3-pT3 positive cells are shown ($n \geq 16$) . Scale bars, 10 μm

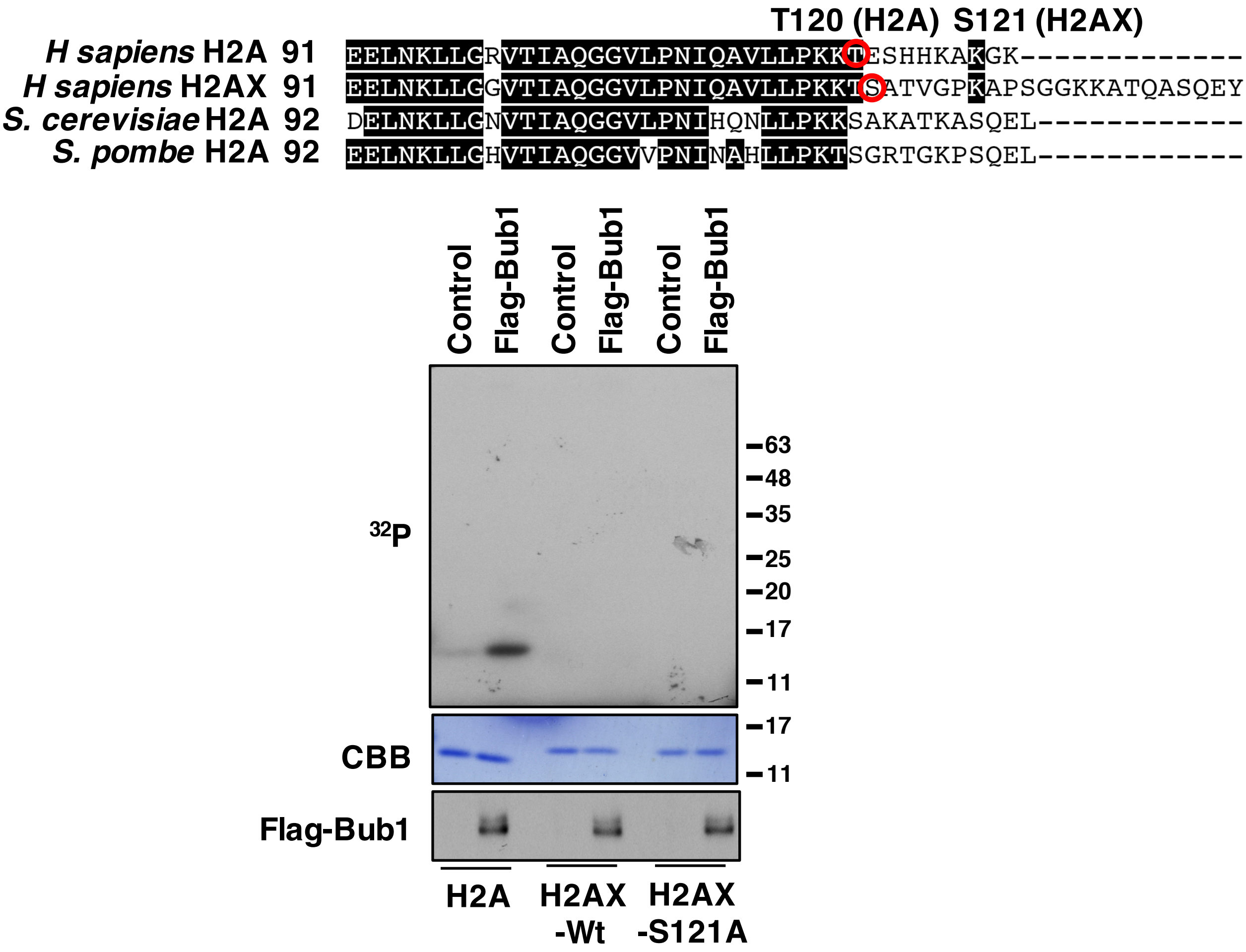

Supplementary Figure 9

Bub 1 phosphorylates H2A, but not H2AX, *in vitro*
Sequence alignment of C-terminal regions of *Homo sapiens* H2A *and* H2AX, and *S. cerevisiae* and *S. pombe* H2A (Top). T120 of H2A and S121 of H2AX are circled in red. Recombinant Bub1 was used for *in vitro* kinase assays with [^{32}P]-ATP using purified human H2A and H2AX-Wt or S121A as substrates (Bottom, upper panels). Purified substrates were subjected to CBB staining (2nd panel) and Flag-Bub1 was subjected to immunoblotting using anti-Flag antibodies (lower panel).

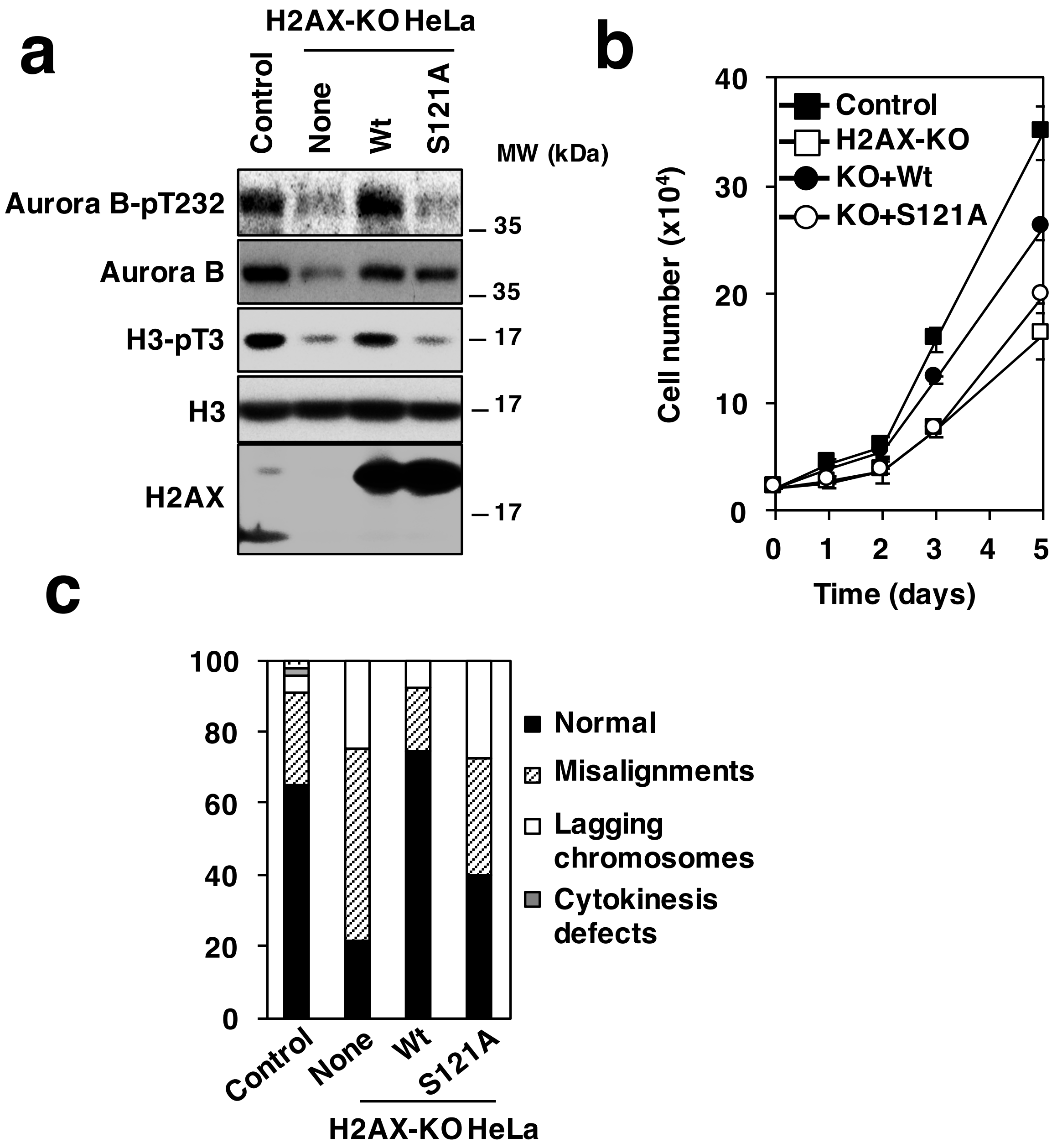

Supplementary Figure 10

Wild-type H2AX, but not the S121 A mutant, rescues impaired cell proliferation and proper chromosome segregation in H2AX-KO HeLa cells.
(a) Control, H2AX-KO, H2AX-KO expressing Flag-H2AX-Wt or S121A HeLa cells were incubated with nocodazole for 12 hrs. After mitotic shake off, cells were collected and their chromatin fractions were subjected to immunoblotting using the indicated antibodies. **(b)** Control, H2AX-KO, or H2AX-KO expressing the Dox-inducible Flag-wild-type (Wt), or S121A mutant HeLa cells were cultured in the presence of doxycyclin (1 μg/ml) and their numbers were counted at the indicated times. Data are shown as means ± SD of at least three independent experiments. **(c)** H2AX-KO HeLa cells with H2B-EGFP expressing Flag-H2AX-WT or S121A were subjected to time-lapse imaging for 24 hrs to monitor chromosome dynamics. Cells showing the indicated phenotypes are quantified (n≥40) .

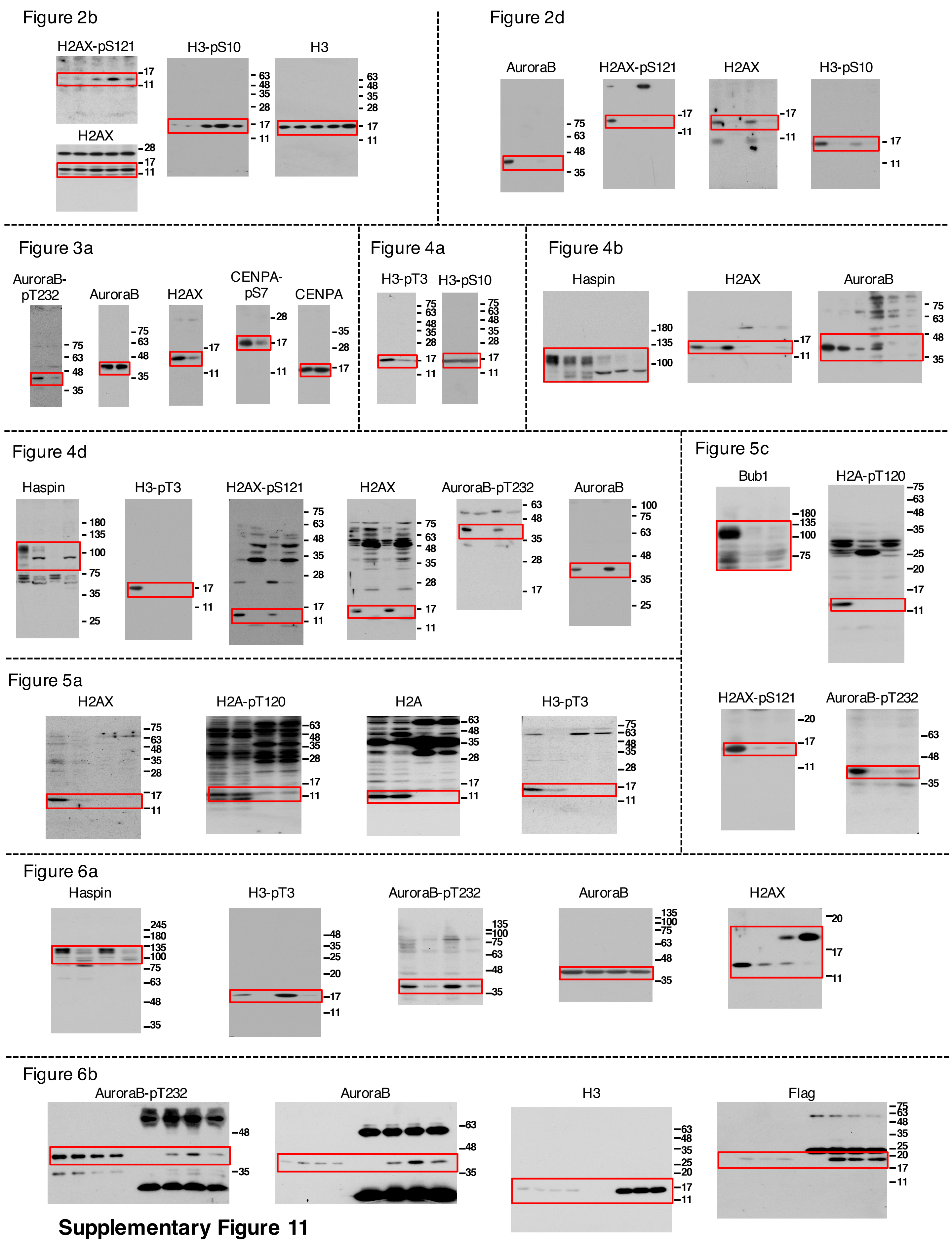

Supplementary Figure 11
Full scans of the most important blots

Supplementary Table 1

Antibodies used in this study

Antibodies	Product number	Dilution for WB	Dilution for IF	Source
H2AX	ab11175	1/2000	1/400	Abcam
H3	ab1791	1/10000		Abcam
H3pT3		1/2000	1/100	Gift of H. Kimura and N. Nozaki
H2AXpS121		1/1000	1/100	Generated in this study
H3pS10	06-570	1/3000		Millipore
AuroraB	BD 611082	1/2000	1/100	BD Biosciences
AuroraB-pT232	600-401-677	1/2000	1/200	Rockland
AuroraB-pT232	cs2914	1/2000		Cell signaling
β-actin	ab6276	1/6000		Abcam
CREST	15-234-0001		1/100	Antibodies Inc.
Haspin	A302-241A	1/2000		Bethyl Laboratories, Inc.
H2ApT120	39391	1/2000	1/1000	Active Motif
H2A	ab18255	1/3000		Abcam
Bub1	ab54893	1/2000		Abcam
Cdc25C	sc327	1/2000		Santa Cruz
GFP	D153-3	1/2000	1/100	MBL
INCENP	cs2786	1/2000		Cell signaling
Survivin	cs2808	1/3000		Cell signaling
Borealin	sc376635	1/2000		Santa Cruz
CENPA	ab13939		1/100	Abcam
CENPA	cs2186	1/2000		Cell signaling
CENPA-pS7	cs2187	1/2000	1/100	Cell signaling
Flag	M2	1/6000		SIGMA

Supplementary Table 2

The target sequences for lentivirus-based shRNAs

Target	Sequence
shH2AX-1	GGGACGAAGCACTTGGTAACA
ahH2AX-2	GACAACAAGAAGACGCGAATC
shAuroraB	GCAGAGAGATCGAAATCCAGG
shHaspin	GCACTCCTCCTCTATGTATTT
shBub1-1	GGAAGTGCCTCATGCTGAAGA
shBub1-2	GCCCAAGACTGAATTTCAATT
